# Using modified aptamers for site specific protein–aptamer conjugations†
†Electronic supplementary information (ESI) available: All experimental details are written as a separate section in the SI materials. These include the synthetic procedures, testing protocols *etc.* They are written in detail and are complete. See DOI: 10.1039/c5sc02631h


**DOI:** 10.1039/c5sc02631h

**Published:** 2015-12-10

**Authors:** Ruowen Wang, Danqing Lu, Huarong Bai, Cheng Jin, Guobei Yan, Mao Ye, Liping Qiu, Rongshan Chang, Cheng Cui, Hao Liang, Weihong Tan

**Affiliations:** a Molecular Sciences and Biomedicine Laboratory , State Key Laboratory for Chemo/Biosensing and Chemometrics , College of Chemistry and Chemical Engineering and College of Biology , Collaborative Innovation Center for Molecular Engineering and Theranostics , Hunan University , Changsha 410082 , China; b Departments of Chemistry and Department of Physiology and Functional Genomics , Center for Research at the Bio/Nano Interface , Shands Cancer Center , UF Genetics Institute and McKnight Brain Institute , University of Florida , Gainesville , Florida 32611-7200 , USA . Email: tan@chem.ufl.edu ; Email: ruowenwang@ufl.edu

## Abstract

Conjugation of DNA to defined locations on a protein surface will be a powerful tool for positioning functional groups and molecules in biological and biomedical studies. However, tagging protein with DNA is challenging in physiological environments, and requires a bioorthogonal approach. Here, we report a chemical solution to selectively conjugate DNA aptamers with a protein by protein–aptamer template (PAT)-directed reactions. Since protein–aptamer interactions are bioorthogonal, we exploit the PAT as a unique platform for specific DNA–protein cross-linking. We develop a series of modified oligonucleotides for PAT-directed reactions and find an F-carboxyl group as a suitable functionality for selective and site-specific conjugation. The functionality is incorporated into aptamers in our F-carboxyl phosphoramidite with an easy synthesis. We also demonstrate the necessity of a linker between the reactive functionality and the aptamer sequences.

## Abstract 2

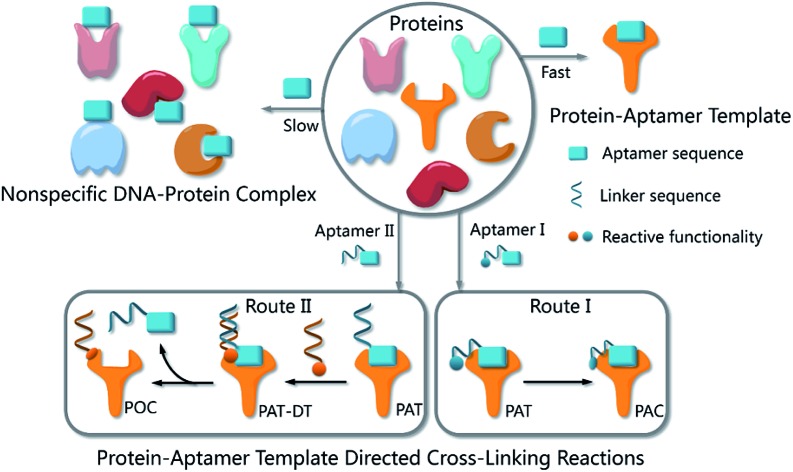
We have developed a new method for the selective conjugation of target proteins at lysine residues through a protein–aptamer template-directed reaction.

## Introduction

Proteins are essential biomolecules of organisms and participate in every cellular process.^1^ To study proteins in their native microenvironments, several methods have been developed to equip proteins with reporter tags *in vivo* for visualization and isolation.^2^,^3^ Recently developed bioorthogonal chemistry provides practical methods to tag proteins with small-molecule probes.^4^–^8^

**Fig. 1:**
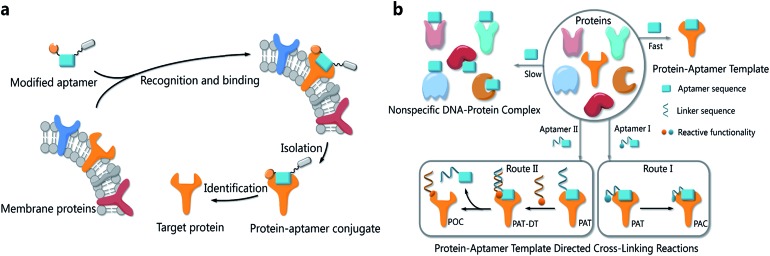
(a) The process of a one-step bioorthogonal reaction for biomarker discovery; (b) general approaches to the preparation of protein–aptamer conjugates (PAC, route I) or protein–oligonucleotide conjugates (POC, route II) by PAT-directed reactions.

The functionalities of small molecules can be incorporated into oligonucleotides by automated synthesis.^9^ Furthermore, oligonucleotides with given sequences created by SELEX^10^,^11^ can recognize target proteins and provide a unique means for manipulation. Therefore, such DNA–protein conjugates possess many exceptional properties with important applications in biomedical diagnostics, bioanalysis and nanobiology.^12^–^14^ Researchers have developed a variety of methods to provide DNA–protein conjugates. In 2012, Famulok *et al.*, for example, employed aptamers modified with reactive functionalities to selectively label target proteins.^15^ Very recently, Gothelf and coworkers applied DNA-templated synthesis to accomplish site-selective labeling of antibodies, transferrin and metal-binding proteins.^16^ As our probe molecules of choice, several DNA aptamers that recognize membrane proteins on cancer cells have been developed.^17^,^18^ Identification of these membrane proteins is important not only because they are potential biomarkers,^19^–^21^ but also because they hold the key to understanding biological processes in cancer cells.^22^,^23^ However, bioorthogonal reactions for aptamer-based biomarker discovery and other applications are challenging when the protein substrates cannot be modified in advance. We had prepared modified TD05 aptamers and managed to identify the target proteins on Ramos cells^24^ by photo-cross-linking,^25^–^27^ while this method didn't work for other aptamers selected by our group. Aptamers modified with aldehyde^28^ were also tried to tag the target membrane proteins. We tested that the modified aptamers still specifically recognize and bind with the target protein on the cell surface, forming a stable complex named as a protein–aptamer template (PAT). PAT-directed cross-linking may provide a platform for bioorthogonal conjugation of aptamers with target proteins. Therefore, we proposed PAT cross-linking as a one-step bioorthogonal reaction to tag, isolate and identify target membrane proteins (Fig. 1a) in 2011, and proposed that many factors should be explored for this type of reaction to establish standard and practical cross-linking protocols for aptamer-based biomarker discovery.^29^ Herein we report a new method for selective conjugation of target proteins at lysine residues through a PAT-directed reaction by aptamers tethered with an F-carboxyl group.

## Results and discussion

Compared with a DNA template, a PAT is more complicated, and it is more difficult to predict the structure; nonetheless, PAT is expected to be a suitable template for directing reactions in a controllable way, as long as the aptamer is well designed. In both physiological environments and buffer solutions, protein–aptamer interactions are bioorthogonal, and aptamers can recognize and bind to their target proteins with high affinity, providing PAT with the requisite orthogonality (Fig. 1b). (On the other hand, a nonspecific DNA–protein complex may be formed, albeit more slowly, at low concentration in the same system.) The resultant PAT promotes a cross-linking reaction to give a protein–aptamer conjugate (PAC, route 1, Fig. 1b) when a reactive functionality (aquamarine circle) is incorporated into the aptamer's 3′- or 5′-end *via* a linker (aquamarine ribbon). PATs may also facilitate the bioorthogonal reaction of the protein with a second oligonucleotide through a PAT–DT complex to give a protein–oligonucleotide conjugate (POC, route II, Fig. 1b). Here we report the development of PAT-directed reactions as a practical strategy to selectively modify proteins with aptamers at a specific site. We evolve a series of modified oligonucleotides that react with proteins. By using an aptamer incorporated with an α,α-*gem*-difluoromethyl carboxyl group (F-carboxyl), we successfully label target proteins at lysine residues. We demonstrate that both the reactive functionality and linker are important factors for PAT-directed reactions.

When a functionalized aptamer binds with its target protein to form a PAT, the aptamer's 3′- or 5′-end may be kept distant from the aptamer's surface in accordance with some crystallographic structures of the protein–aptamer complex.^30^–^32^ In addition, the reactive functional group tethered to an aptamer without linker may be kept distant from lysine residues. Therefore, a linker between the reactive functional group and the aptamer sequence is necessary for site-specific conjugation, allowing the functional group to reach out to and cross-link with the site on the protein surface. To verify our hypothesis and explore a proper linker for PAT-directed reactions, we have synthesized a series of aptamers tethered with an aldehyde *via* different linkers (see Table S1 in ESI for detailed sequences†). Aldehyde functionality can be readily incorporated into oligonucleotides using a RNA cpg^33^ or commercially available 5-aldehyde-modifier phosphoramidite.^34^

**Fig. 2:**
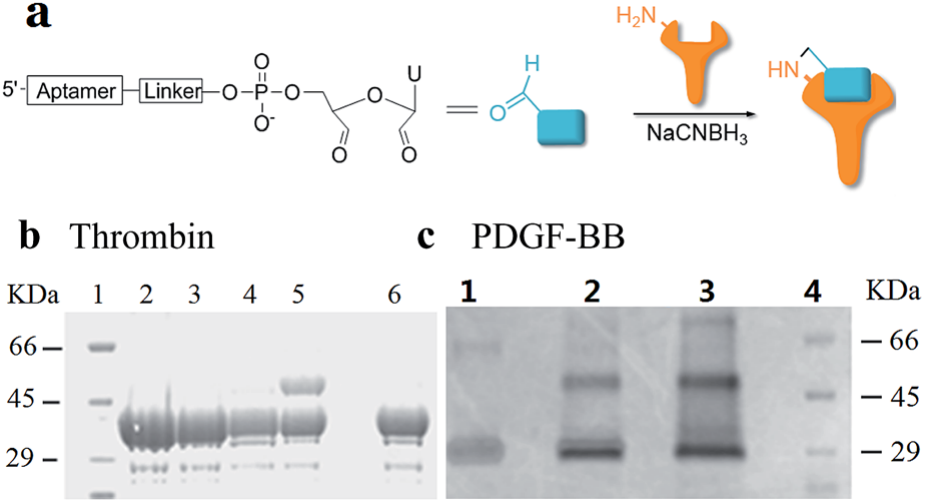
Cross-linking of proteins with aldehyde-modified aptamers. (a) Structure of an aldehyde-modified aptamer and its reaction with protein. (b) SDS PAGE analysis of thrombin reacted with aptamers (5X) with different linkers: lane 1: molecular weight marker; lane 2: thrombin + **A-1**; lane 3: thrombin + **A-2**; lane 4: thrombin + **A-3**; lane 5: thrombin + **A-4**; lane 6: thrombin. (c) SDS PAGE analysis of PDGF-BB reacted with aptamers with 8T linkers (5X) at different concentrations: lane 1: PDGF-BB; lane 2: PDGF + **A-5** (5X); lane 3: PDGF-BB + **A-5** (20X); lane 4: molecular weight marker.

Using RNA-CPG, *e.g.* U-CPG, a series of thrombin aptamers^35^ were prepared with a vicinal diol group at the 3′-end. Oxidation of the vicinal diol with NaIO_4_ led to aldehyde-modified aptamers (Fig. 2a). The PAT-directed reactions of aldehyde-modified aptamers were initially carried out in 10–20 μL buffer (pH 7) in the presence of NaCNBH_3_ at 4 °C. The reactions of the aptamers with different linkers were compared, and the results were determined by SDS PAGE gel (Fig. 2). Based on the gel results, no reaction was detected between thrombin and aptamer **A-1** (with no linker, Fig. 2b, entry 2), **A-2** (with a PEG linker, Fig. 2b, entry 3), or **A-3** (with a 3T linker, Fig. 2b, entry 4). Fortunately, however, the reaction of aptamer **A-4** (with an 8T linker, Fig. 2b, entry 5) with thrombin did give the cross-linked product in moderate yield. These results showed the importance of a linker for PAT-directed reactions. To verify if the 8T linker would be applicable to other PAT-directed reactions, another protein–aptamer tethered to an aldehyde *via* an 8T linker (PDGF-BB aptamer,^36^ see Table S1 in ESI for detailed sequences†) was prepared. The reaction of the PDGF-BB with **A-5** also gave the cross-linked conjugate in moderate yield, as expected (Fig. 2c, entry 2), and the yield was improved when the concentration of aptamer was increased (Fig. 2c, entry 3).

**Fig. 3:**
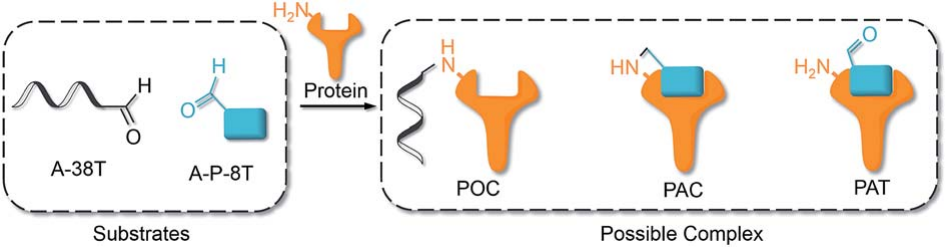
Interactions of aldehyde-modified aptamers with protein.

The interaction of an aptamer with its target protein is highly selective, and high-affinity binding results in the rapid formation of a PAT (Fig. 1b). In contrast, nonspecific interactions of aptamers with other proteins may slowly lead to formation of an unstable protein–oligonucleotide complex (POC, Fig. 1b) at low percentages. PATs promote bimolecular reactions by pulling two reactants close to each other in a manner similar to that of an intramolecular reaction. We propose that PAT-directed conjugation is a bioorthogonal reaction based on the hypothesis that protein–aptamer cross-linking proceeds mainly within the template. However, nonspecific conjugation may also proceed effectively to provide a protein–oligonucleotide conjugate (POC, Fig. 3), without the promotion by a PAT, when the aptamer is modified with a functionality that is highly reactive with proteins. Therefore, the reactivity of modified aptamers is another critical component ensuring the selectivity of PAT-directed conjugation.

An aldehyde group represents a functionality that is highly reactive with protein and has been incorporated into oligonucleotides for the preparation of nonspecific DNA–protein conjugates. Hence, the interactions of aldehyde-modified oligonucleotides with proteins may result in the formation of three different complexes, as shown in Fig. 3: protein–oligonucleotide conjugate (POC, nonspecific), protein–aptamer conjugate (PAC, specific), and protein–aptamer template (PAT, specific). The reduction reaction of a PAT with NaCNBH_3_ provides a PAC as the product if the linker tethering the aldehyde with the aptamer is appropriate (as we demonstrate in Fig. 2a).

**Fig. 4:**
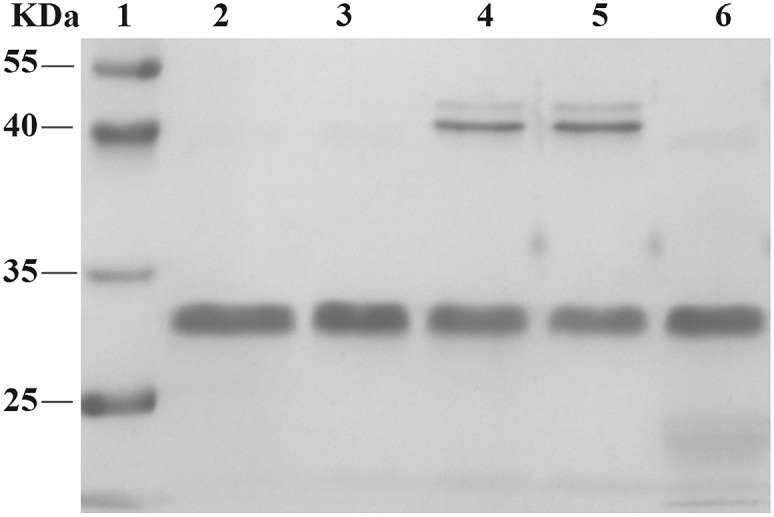
SDS-PAGE analysis of control reactions. lane 1: molecular weight marker; lane 2: PDGF-BB; lane 3: PDGF-BB, **A-6**, **A-7**; lane 4: PDGF-BB, **A-6** (20X); lane 5: PDGF-BB, **A-5**, **A-7** (5X); lane 6: PDGF-BB, **A-5**, without addition of NaCNBH_3_.

To explore the specificity of aldehyde-modified aptamers, we compared the reaction of **A-5** with the control reaction of **A-6** (see Table S1 in ESI for detailed sequences and reaction conditions†). As shown in Fig. 4, the reaction of **A-6** (20X) gave POCs in moderate yield (lane 4, Fig. 4), comparable to that of **A-5** (5X, Fig. 3c, lane 2). Since nonspecific aldehyde-modified oligonucleotides still react with proteins without the promotion of a PAT, the results confirm the hypothesis that the reactivity of a modified aptamer is critical for the selectivity of a PAT-directed reaction. Interestingly, this nonspecific reaction can be inhibited by the addition of aptamer **A-7** (without aldehyde-modification, lane 3, Fig. 4). We infer that **A-7** prevents the nonspecific interaction of **A-6** with PDGF-BB and their cross-linking when the protein is binding with **A-7**. In the presence of **A-7**, the reaction of **A-5** and PDGF-BB still gave the cross-linked PAC in a moderate yield (lane 5, Fig. 4) because protein–aptamer binding is a reversible interaction and **A-5** competes equally with **A-7** to form the template. Not surprisingly, no PACs were observed for the reaction of **A-5** with PDGF-BB without the addition of NaCNBH_3_ (lane 5, Fig. 4). The results indicate that an aldehyde-aptamer may still be suitable for the bioorthogonal labeling of membrane proteins on cell surfaces because the aptamers that are nonspecifically binding with other proteins can be washed away before the addition of NaCNBH_3_, eliminating POC formation.

On the observation of the partial selectivity of aldehyde-modified aptamers, we had been looking for a proper functional group to realize both bioorthogonal and site-specific modification of proteins for general PAT-directed reactions. A carboxyl group is coupled with an amine group directly to form peptide bonding as a result of enzyme catalysis in the biosynthesis of proteins, but the carboxyl has to be activated for efficient coupling in peptide synthesis.^37^ The reactivity of the carboxyl group is tunable, and the introduction of an adjacent electron-withdrawing group (EWG) can enhance reactivity with the nucleophilic amine.

**Fig. 5:**
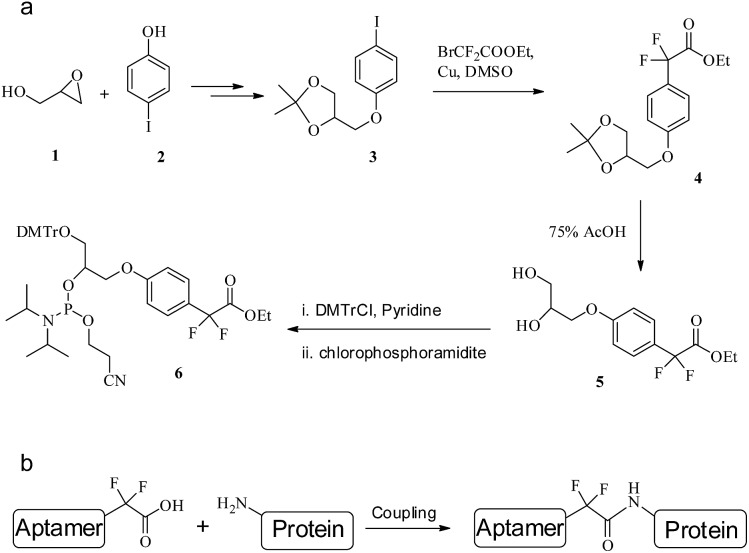
(a) The synthetic route to F-carboxyl phosphoramidite **6**. (b) The coupling of an F-alkyl aptamer with protein.

Perfluoroalkyl (F-alkyl) groups are EWGs with chemically inert properties. α,α-*gem*-Difluoromethyl (F-carboxyl) is the smallest perfluoroalkyl group in size, and it has been verified that F-carboxyl is a moderate amine-reactive electrophile.^38^ We extrapolated that nonspecific reactions of F-carboxyl-functionalized aptamers with the amine of lysine residues in aqueous solution might be negligible, but that intramolecular reactions within the PAT could be efficient in view of the fact that the F-carboxyl group is assembled close to the amine group. Thus, the cross-linking reaction of an F-carboxyl aptamer with the target protein could efficiently give a site-specific PAC with selectivity. Moreover, the linker between the aptamer and protein is an amide bond (Fig. 5b), which can be enzymatically dissociated. Therefore we have designed and synthesized an F-carboxyl phosphoramidite for convenient preparation of an F-carboxyl aptamer.

As shown in Fig. 5a, the synthesis of F-alkyl carboxyl phosphoramidite **6** starts with the coupling of commercially available glycidol **1** and 4-iodophenol **2**.^39^ Cross-coupling ethyl bromodifluoroacetate with the acetonide-protected precursor **3** provides the α,α-*gem*-difluoromethyl derivative **4**.^40^ Removal of the acetonide group gives diol **5** in high yield, where the two hydroxyl groups are protected by DMTr and the phosphoramidite group, respectively,^41^ giving phosphoramidite **6** (see ESI for details†).

**Fig. 6:**
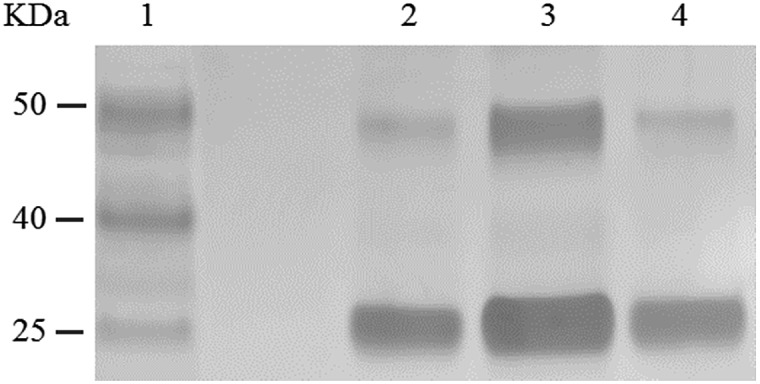
SDS-PAGE analysis of PDGF-BB reacted with F-carboxyl oligonucleotides. Lane 1: molecular weight marker; lane 2: PDGF-BB; lane 3: PDGF-BB, **F-1**; lane 4: PDGF-BB, **F-2**.

From phosphoramidite **6**, we prepared F-carboxyl aptamer **F-1** and oligonucleotide **F-2** as control (see Table S1 in ESI for detailed sequences†). A PAT-directed reaction of F-carboxyl aptamer **F-1** with PDGF-BB gave a cross-linked PAC product with a yield (lane 3, Fig. 6) comparable to that of aldehyde-modified aptamers. While in a nonspecific reaction of F-carboxyl oligonucleotide **F-2** that was carried out in similar conditions, no cross-linked product was detected by SDS-PAGE (lane 4, Fig. 6). The PAC band in lane 3 was cut and sent for mass analysis, and the results confirmed the structure of the conjugate.

**Fig. 7:**
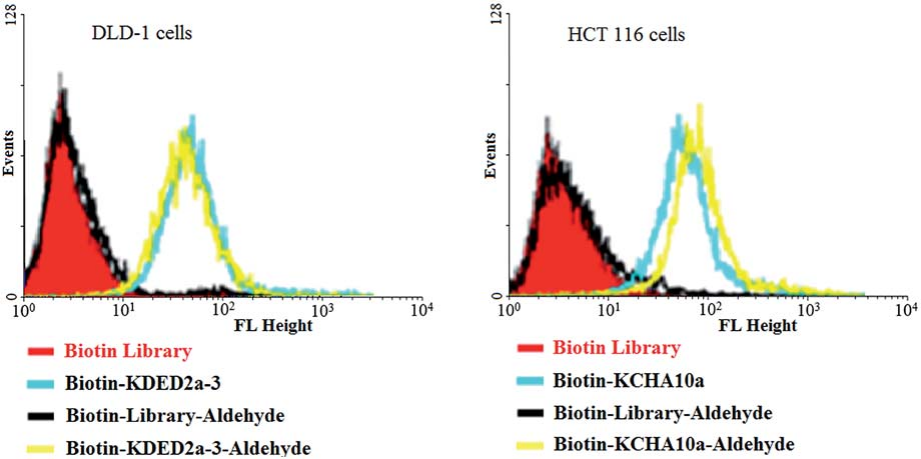
Aptamers modified with an aldehyde at 3′ and biotin at 5′ could still bind specifically to the target cells.

When reactive functionalities are tethered to the 3′- or 5′-end of the cell-aptamer sequence, they may affect the binding affinity. Accordingly, we evaluated the specific recognition and binding ability of biotinylated aptamers KDED2a-3 and KCHA10a^42^ modified with an aldehyde at the 3′-end (biotin-KDED2a-3-aldehyde and biotin-KCHA10-aldehyde; see Table S1 in ESI for detailed sequences†). For visualization, each complex was combined with a streptavidin-PE-Cy5.5 (phycoerythrin) dye for the cell binding assay. In this assay, flow cytometry was used to monitor the fluorescence intensities of cells, with aptamer biotin-KDED2a-3 and biotin-KCHA10a as a positive control and random sequences (Biotin Library and Biotin-Library-aldehyde) as a negative control. As shown in Fig. 7, the fluorescence intensities of cells incubated with an aldehyde-modified control (Biotin-Library-aldehyde) are as weak as those bound with Biotin Library, excluding the possibility of nonspecific binding caused by aldehyde. The fluorescence intensities of DLD-1 cells and HCT 116 cells bound with respective aldehyde-modified aptamers were comparable to those bound with positive controls, indicating that these modified aptamers still bind specifically with their target proteins.

Similarly, retention of selectivity and binding affinity were also observed for F-carboxyl aptamer (**F-3**). The binding affinity assay indicates the formation of PATs.

## Summary

In conclusion, we have developed a new method for the selective conjugation of target proteins at lysine residues through a PAT-directed reaction. This was achieved by an aptamer tethered with an F-carboxyl group *via* an 8T linker. PATs promote the cross-linking of proteins and aptamers by pulling together the F-carboxyl group and the amine of a lysine residue. We evolved a series of oligonucleotides tethered with amine-reactive functionalities *via* different linkers. Comparing the reactions of aldehyde-modified aptamers with different linkers, we optimized 8T as an appropriate linker between the aptamer sequence and the reactive functionality. On the observation of the partial selectivity of the aldehyde-modified aptamer, we designed and developed a novel phosphoramidite to incorporate the F-carboxyl group as a proper functional group for PAT-directed reactions. We found that nonspecific reactions of F-carboxyl oligonucleotides with proteins are negligible, while, on the other hand, a PAT-directed reaction of the F-carboxyl aptamer provides a cross-linked product efficiently. Flow cytometry studies confirmed that aptamers retain their specific recognition and binding abilities, even when they are modified with reactive functionalities. It has been illustrated that a PAT is a suitable template to direct reactions in a controllable way. We believe that this method will be especially valuable for cell membrane protein imaging and isolation and other related biological studies.

## Conflict of interest

The authors declare no competing financial interests.

## Supplementary Material

Supplementary information
