# Supplementary material for: Using modified aptamers for site specific protein–aptamer conjugations
Source: Chem Sci. 2015 Dec 10;7(3):2157–61. doi: 10.1039/c5sc02631h (PMC5201207; doi:10.1039/c5sc02631h)
Supplement: Supplementary file 1 [file SC-007-C5SC02631H-s001.pdf]

Electronic Supplementary Material (ESI) for Chemical Science.
This journal is © The Royal Society of Chemistry 2015

Supporting Information

Using modified aptamers for site specific protein-aptamer conjugations

Ruowen Wang[†‡§]*, Danqing Lu[†§], Huarong Bai[†§], Cheng Jin[†], Guobei Yan[†], Mao Ye[†], Liping Qiu[†‡], Rongshan Chang[†,] Cheng Cui[‡], Hao Liang[†] and Weihong Tan[†‡]*

Table of Contents

Synthetic Protocols……………………………………………….…..…...……...S3

Scheme S1: The synthetic routes to phosphoramidite **6**…….………..….......……S3

Synthesis of compounds **4-7**………………..……………….…………......……..S3

Synthesis and purification of oligonucleotides..…………………….…...….……...S4

Table S1: Detailed sequence information of oligonucleotides…………….……S4

Protocols for PATCL reactions..……………...………………….…………..……S5

Cell culture…………………………………………………….……....……......S6

Binding tests ……………………………...……………………..……..……..……S6

References……………………………...…………………….……..……......……S7

Synthetic protocols

Unless otherwise noted below, all commercially available reagents and solvents were purchased from Sigma Aldrich and used without further purification. ^{1}H NMR (TMS as the internal standard) and ^{19}F NMR spectra ($CFCl_3$ as the outside standard and low field positive) were recorded on a Bruker AM300 or Bruker AM400 spectrometer. Chemical shifts (δ) are reported in ppm, and coupling constants (J) are in Hertz (Hz).

Scheme S1. The synthetic routes to phosphoramidite **6**.

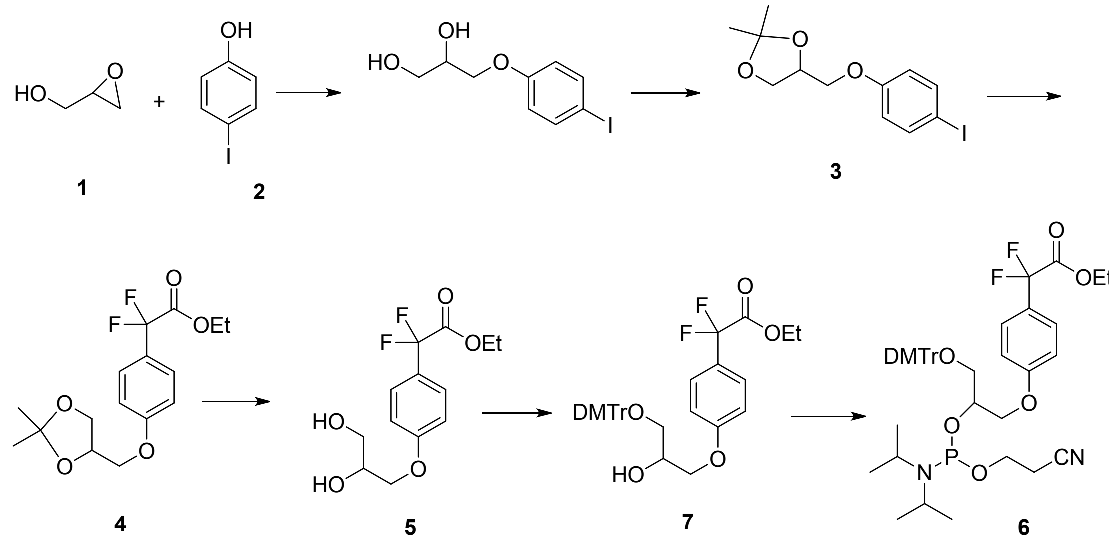

Synthesis of compound **4**

Precursor **3** was synthesized from commercially available glycidol 1 and 4-iodophenol 2 in two steps following established methods.[1,2] In an atmosphere of Ar, ethyl bromodifluoroacetate (0.50 ml, 4.0 mmol) was added to a suspension of activated Cu power (706 mg, 11.2 mmol) and acetonide-protected glycerol ether 3 (1.34g, 4.0 mmol) in DMSO (20mL), and the mixture was stirred at 55 ^{o}C for 9h. The mixture was poured into a mixture of ice and saturated NH_4Cl, followed by extraction with Et_2O. The organic layer was washed with saturated NH_4Cl and saturated NaCl aqueous solution, dried over Na_2SO_4, and concentrated *in vacuo*. The residue was purified by flash chromatography to give compound **4** (0.94 g, 71 % yield): ^{1}H NMR (300 MHz, $CDCl_3$) δ 7.50 (d, J=7.8 Hz, 2H), 7.05 (d, J=7.8 Hz, 2H), 3.97-4.27 (m, 5H), 3.35 (d, J=11.1 Hz, 2H), 1.40 (s, 6H), 1.24 (t, J=7.2 Hz, 3H); ^{19}F NMR (282 MHz, CD_3OD) δ -104.56; MS (ESI+): m/z 331.13 (Calculated M+H: 331.13).

Synthesis of compound **5**

A mixture of compound **4** (0.66g, 2.0 mmol) and 75% aqueous AcOH (10mL) was stirred at 50 ^{o}C for 3h. The solvent was removed *in vacuo*, and the resultant residue was dissolved in EtOAc (15 mL) and washed with aq. $NaHCO_3$ (5mL). The aqueous layer was extracted with EtOAc (10 mL), and the combined organic layer was dried over Na_2SO_4 and concentrated *in vacuo*. The residue was purified by flash chromatography to afford compound **5** (0.54 g, 93 % yield): ^{1}H NMR (400 MHz, CD_3OD) δ 7.49 (d, J=8.1 Hz, 2H), 7.04 (d, J=8.1 Hz, 2H), 4.26 (m, 2H), 3.98-4.11 (m, 3H), 3.66 (s, 2H), 3.32 (d, J=11.1 Hz, 2H), 1.25 (t, J=7.2 Hz, 3H); ^{19}F NMR (282 MHz, CD_3OD) δ -106.92; MS (ESI+): m/z 291.10 (Calculated M+H: 291.10).

Synthesis of compound **7**:

To a solution of compound **5** (0.44g, 1.5 mmol) in anhydrous pyridine (10 mL) was added DMTrCl (0.68 g, 2.0 mmol), and the reaction was stirred overnight. The reaction solution was concentrated, and the residue was purified by flash chromatography to afford compound **7** (0.73 g, 82 % yield): ^{1}H NMR (300 MHz, acetone-d6) δ 7.48-7.55 (m, 4H), 7.25-7.36 (m, 7H), 7.06 (d, J=7.8 Hz, 2H), 6.85 (d, J=7.5 Hz, 4H), 4.03-4.32 (m, 5H), 3.76 (s, 6H), 3.28 (s, 2H), 2.94 (s, 1H) , 1.26 (t, J=7.2 Hz, 2H); ^{19}F NMR (282 MHz, acetone-d6) δ -102.56; MS (ESI+): m/z593.23 (Calculated M+H: 593.23).

Synthesis of phosphoramidite **6**

To a solution of compound **7** (0.59g, 1.0 mmol) in anhydrous DCM (10 mL) was added DIEA, followed by chlorophosphoramidite (310 mg, 1.3 mmol) at 0°C. The mixture was allowed to warm to RT and was stirred for 0.5-1 h. The reaction was then diluted with 50 mL of DCM, washed with saturated $NaHCO_3$ solution, and saturated saline solution. The organic phase was dried over Na_2SO_4 and then concentrated. The residue was purified by flash chromatography to afford phosphoramidite **6** (0.67 g, 85 % yield): ^{1}H NMR (300 MHz, acetone-d6) δ 7.44-7.53 (m, 4H), 7.26-7.34 (m, 7H), 7.05-7.10 (m, 2H), 6.84-6.87 (m, J=7.5 Hz, 4H), 4.10-4.39 (m, 5H), 3.76 (s, 6H), 3.56-3.88 (m, 2H), 3.29-3.40 (m, 2H), 2.86 (m, 2H), 2.72-2.74 (m, 1H), 2.60-2.63 (m, 1H), 1.05-1.28 (m, 15H); ^{19}F NMR (282 MHz, acetone-d6) δ: -102.12, -102.16; ^{31}P NMR(acetone-d6) δ: 149.55, 149.21.

Synthesis and purification of oligonucleotides:

All DNA synthesis reagents were purchased from Glen Research. Oligonucleotides were synthesized on an ABI 3400 DNA synthesizer with reagents purchased from Glen Research (Sterling, VA, USA). The DNA sequence was uploaded into a DNA synthesizer online, and the synthesis protocol was set up according to the requirements specified by the reagents' manufacturers. Following on-machine synthesis, all oligonucleotides, except F-carboxyl oligonucleotides, were deprotected and cleaved from CPG by incubating with 2.5 mL ammonium hydroxide for 30 min at 65°C in a water bath. F-carboxyl oligonucleotides were deprotected and cleaved from CPG by incubating with 2.5 mL sodium hydroxide solution (0.4 M) in methanol/water (4/1) at r.t. for 12h. The cleaved DNA product was transferred to a 15 mL centrifuge tube and mixed with 250 µL 3.0 M NaCl and 6.25 mL ethanol, after which the sample was placed into a freezer at -20°C for ethanol precipitation. Afterwards, the DNA product was spun at 4000 rpm at 4°C for 30 minutes. The supernatant was removed, and the precipitated DNA product was dissolved in 500 µL DNA grade water for HPLC purification using a cleaned Alltech C18 column on a Varian Prostar HPLC. The collected DNA product was dried and detritylated by dissolving and incubating in 200 µL 80% acetic acid for 20 minutes. The detritylated DNA product was mixed with 400 µL ethanol and dried by a vacuum dryer. The purified probe was quantified by determining the UV absorption at 260 nm, after which the probe was dissolved in DNA grade water and stored in the freezer at -20 ^{o}C for future experiments.

Table S1. Detailed sequence information of oligonucleotides[a].

Name	Sequence [1]
A-1	5'-GGTTG GTGTG GTTGG U

A-2	5'-GGTTG GTGTG GTTGG **PEG**U
A-3	5'-GGTTG GTGTG GTTGG **TTT**U
A-4	5'-GGTTG GTGTG GTTGG **TTTTT TTT**U
A-5	5'-CAGGC TACGG CACGT AGAGC ATCAC CATGA TCCTG **TTTTT TTT**U
F-1	5'-F**TTTT TTTT** CAGGC TACGG CACGT AGAGC ATCAC CATGA TCCTG
A-6	5'-TTTTT TTTTT TTTTT TTTTT TTTTT TTTTT TTTTT TTTU
A-7	5'-CAGGC TACGG CACGT AGAGC ATCAC CATGA TCCTG **TTTTT TTT**
F-2	5'-FTTTT TTTTT TTTTT TTTTT TTTTT TTTTT TTTTT TTTT
Biotin-KDED2a-Aldehyde	5'-Biotin-TGCCC GCGAA AACTG CTATT ACGTG TGAGA GGAAA GATCA CGCGG GTTCG TGGAC ACGG**T TTTTT TTT**U
Biotin-KCHA10-Aldehyde	5'-Biotin-ATCCA GAGTG ACGCA GCAGG GGAGG CGAGA GCGCA CAATA ACGAT GGTTG GGACC CAACT GTTTG GACAC GGTGG CTTAG **TTTTT TTTT**U
F-3	5'-F**TTT TTTTT**-TGCCC GCGAA AACTG CTATT ACGTG TGAGA GGAAA GATCA CGCGG GTTCG TGGAC ACGGT
Biotin-Library	5'-Biotin-(N)$_n$, n=69 or 90
Biotin-Library-Aldehyde	5'-Biotin-(N)$_n$U, n=69 or 90

[a] Letters in bold indicate the sequences of linkers. F represents the unit synthesized from phosphoramidite **6**

Synthesis of oligonucleotides modified with aldehyde at the 3'-end.

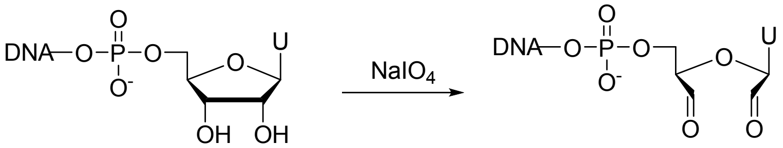

For the introduction of the aldehyde group, commercially available RNA CPG U was used for DNA synthesis. To a solution of purified oligonucleotides (50 nmol) in 125 µL H_2O was added 15 µL 1 M sodium phosphate buffer (pH 5.4) and 10 µL 50 mM $NaIO_4$. The reaction mixture was kept in the dark for 6 hours at 4 ^oC. Excess $NaIO_4$ was quenched with 10 µL 55 mM Na_2SO_3. The aqueous solvent was removed by lyophilization, and the residue was desalted to provide the desired DNA modified with aldehyde.

Synthesis of oligonucleotides modified with F-carboxyl group.

The F-carboxyl group could be automatically incorporated into oligonucleotides from our phosphoramidite **6** by the DNA synthesizer. Since the F-carboxyl group is a type of activated carboxyl group, sodium hydroxide was chosen instead of ammonium hydroxide for the cleavage and deprotection steps. Other synthesis and purification steps of F-carboxyl oligonucleotides are similar to those for general oligonucleotides.

Protocols for PATCL reaction of aldehyde-modified substrates

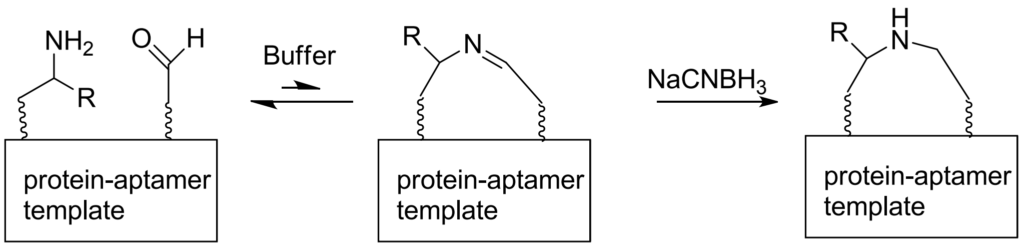

To an 11 μL solution of 0.01 nmol of protein (Thrombin or PDGF-BB) and 0.05 nmol aptamer (0.2 nmol for 20X cross-link reaction) in 0.1 M sodium phosphate buffer was added 1 μL 0.5 M $NaCNBH_3$. The reaction mixture was left in the dark at 4°C for 6-8 hours. The protein-aptamer cross-linking formation was assessed by polyacrylamide gel electrophoresis (12%) (SDS-PAGE). To the reaction solution was added 4X loading buffer, and the mixture was heated to 90°C for 4 minutes and used in SDS-PAGE gel directly. The resultant gel was stained with SilverQuest Silver Staining Kit (Invitrogen and Shanghai Sangon).

Protocol for PATCL reaction of F-carboxyl substrates

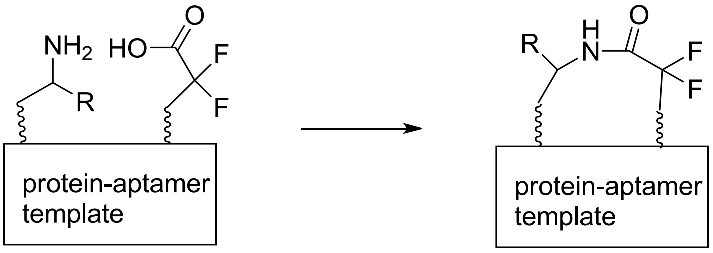

The solution of 0.01 nmol of protein (PDGF-BB) and 0.20 nmol aptamer in 11 μL 0.1 M sodium phosphate buffer was left in the dark at 4°C for 6-8 hours. The protein-aptamer cross-linking formation was assessed by polyacrylamide gel electrophoresis (12%) (SDS-PAGE). To the reaction solution was added 4X loading buffer, and the mixture was heated to 90°C for 4 minutes and used directly in SDS-PAGE gel. The resultant gel was stained with SilverQuest Silver Staining Kit (Invitrogen).

Cell culture

Colon cancer cell lines DLD-1 (Dukes' type C colorectal adenocarcinoma) and HCT 116 (colorectal carcinoma) were purchased from American Type Cell Culture (Manassas, VA). DLD-1 cells were maintained in culture with RPMI-1640 containing 10% heat-inactivated FBS (Invitrogen) and 100 IU mL^{-1} penicillin-streptomycin (Cellgro). HCT 116 cells were maintained in McCoy's 5A culture medium containing 10% heat-inactivated FBS and 100 Units mL^{-1} penicillin–streptomycin. All cultures were incubated at 37 °C under a 5% CO_2 atmosphere.

Each colon cancer cell line was grown as an adherent monolayer in 100 mm X 20 mm culture dishes to >95% confluence. Cells were washed in the dish with washing buffer (WB) (4.5 g L^{-1} glucose and 5 mM $MgCl_2$ in Dulbecco's PBS with $CaCl_2$, Sigma), dissociated by trypsin treatment (2 min) and seeded into culture dishes at low concentration. Within 24 hours of seeding, cells were briefly trypsinized (30–45 s) for capture studies.

Binding Test

To demonstrate the specific targeting abilities of aldehyde-modified cancer cell aptamers (Biotin-KDED2a-3-Aldehyde and Biotin-KCHA10-Aldehyde) towards DLD-1 and HCT116 cell lines, an aptamer binding test was performed by flow cytometry using a FACScan cytometer (Becton Dickinson Immunocytometry Systems, San Jose, CA, USA). A random sequence (Biotin-Library-Aldehyde) was used as a negative control. Streptavidin-PE conjugate was used to monitor the fluorescence intensities of cells. A green laser at 488 nm with different excitation voltages (650, 700, and 750 V) was used as the excitation source. Samples containing DLD-1 or HCT 116 cells with a concentration of 10^6 cells/ mL were incubated with the desired concentrations of aptamers on ice in a 200 μL volume (0.25 mM) of binding buffer for 30 min. The cells were then washed three times with 1000 μL washing buffer, resuspended in 200 μL binding buffer, and subjected to flow cytometry analysis by counting 10000 events. Data were analyzed with WinMDI software. The remaining sample after flow cytometry analysis was then directly subjected to confocal fluorescence microscopy.

References:

1. Bredikhina, Z. A.; Savel'ev, D. V.; Bredikhin, A. A. *Russ. J. Org. Chem.* **2002**, *38*, 213-219.
2. Tan, X.; Kong, L.; Dai, H.; Cheng, X.; Liu, F.; Tschierske, C. *Chem. Eur. J.* **2013**, *19*, 16303-16313.
